# Curative treatment in a patient with gastric cancer stage IV: a case report

**DOI:** 10.12688/f1000research.1-34.v1

**Published:** 2012-10-19

**Authors:** Andreas Diel, Ernst Rodermann, Hans-Friedrich Kienzle, Denis Meuthen, Ingo Meuthen

**Affiliations:** 1 Practice Network Rhein-Sieg, Hematology and Oncology, Troisdorf / Bonn / Bad Honnef, Germany; 2 Department of Hematology and Oncology, Kliniken der Stadt Köln gGmbH, Krankenhaus Holweide, Germany; 3 Clinic of Surgery, Kliniken der Stadt Köln gGmbH, Krankenhaus Holweide, Germany; 4 Institute for Evolutionary Biology and Ecology, University of Bonn, Bonn, Germany; 5 Practice of Internal Medicine, Hematology and Oncology, Cologne, Rodenkirchen, Germany

## Abstract

A 39-year old patient with gastric adenocarcinoma stage IV failed to respond to preoperative chemotherapies containing 5-FU and cisplatin as well as 5-FU and irinotecan. After third-line chemotherapy with two cycles of docetaxel and cisplatin we confirmed a clinical partial response. A complete histologically confirmed remission was detected after complete resection of the tumor. Following two postoperative cycles of docetaxel and cisplatin, the tumor is still in complete remission after more than eight years.

## Case report

**Figure 1.**
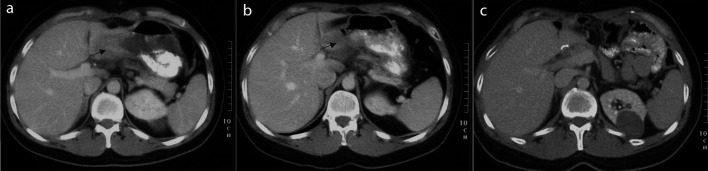
Abdominal CT scans. **a**) before treatment December 2003 **b**) after partial remission May 2004 **c**) after complete remission November 2005.

In November 2003, a 39 year old male patient was diagnosed with a locally advanced gastric adenocarcinoma. The diagnosis and tumor stage were confirmed by gastrocopy, endoscopic ultrasound (EUS = u-staging), computer tomography (CT) scans of the thorax/abdomen and a bone scintigraphy. Gastroscopic biopsies revealed a Helicobacter pylori-negative gastric adenocarcinoma, diffuse type G3. According to the TNM/UICC staging system the carcinoma stage was cT4N3M0 or stage IV, respectively ^1^. We found an invasion of the colon transversum and more than sixteen significantly enlarged lymph nodes ( Figure 1a). We did not test the Her-2-neu status in 2003. Multdisciplinary treatment planning included preoperative chemotherapy followed by operation and postoperative chemotherapy in curative intention. The patient suffered from a severe hypertensive cardiomyopathy, thus he was not eligible for an anthracycline containing chemotherapy.

After six weeks on PLF (cisplatin 50 mg/m ^2^ every two weeks, weekly folinic acid 500 mg/m ^2^ and 5-FU 2000 mg/m ^2^/24h), we found no response. We changed to the FLI-regiment (weekly folinic acid 500 mg/m ^2^, 5-FU 2000 mg/m ^2^/24h, irinotecan 80 mg/m ^2^), there was no response after six weeks as well. After applying two cycles of DC (docetaxel 50 mg/m ^2^ d 1 + 15, cisplatin 50 mg/m ^2^ d 1 + 15, every four weeks), we detected a partial remission on endoscopy and abdominal CT ( Figure 1b). In June 2004, a gastrectomy with a D2 lymph node dissection was performed resulting in a R0 status and a pathologically confirmed complete remission (CR). The patient was in good postoperative condition, he tolerated two further postoperative cycles of DC well without more than grade-2-toxicity. In September 2004, the patient underwent a laparotomy due to an acute bowel obstruction caused by adhesive bands after previous abdominal surgery. No carcinoma was detected macroscopically or histologically ( Figure 1c). Up to this day, more than thirteen years after the diagnosis, the tumor is still in CR. The patient’s quality of life is not compromised in any way (ECOG performance status 0).

Using the TNM/UICC-classification of gastric cancer 2010, our patient would be classified as stage cT4bN3bM0, stage IIIc ^2^.

## Discussion

The 5-year median overall survival rate for stage III and IV patients receiving perioperative chemotherapy is 39.1% and 5.2% after surgical resection alone ^3– 5^. Since 2010, the standard of care in locally advanced gastric cancer (uT3-T4) is three praeoperative cycles of an anthracycin containing polychemotherapy like epirubicine, cisplatin and 5-FU (ECF) followed by three postoperative cycles. This kind of treatment improved 5-year survival from 23% with surgery alone to 36.3% ^6, 7^. There are no sufficient data from randomised phase III studies to strongly recommend perioperative chemotherapy for patients with uT2 carcinomas ^8^.

Two preoperative cycles of PLF seem to be as effective as preoperative ECF ^9, 10^. In advanced gastric cancer, polychemotherapy offers a survival advantage over single-agent therapy. Comparing 5-FU/cisplatin-containing regiments with versus without anthracyclins and 5-FU/anthracyclin-containing combinations with or without cisplatin there is a significant survival benefit for 5-FU + anthracyclins + cisplatin. In this analysis, secondary resectability in locally advanced disease was not reported ^11^.

In metastatic gastric adenocarcimomas and adenocarcinomas of the gastrooesophaeal junction 5-FU can be substituted by capecitabine without compromising the results, so capecitabine may be used instead of 5-FU in the perioperative setting in combination with cisplatin (XP) or with epirubicine and cisplatin (ECX) ^12– 14^. In metastatic disease oxaliplatin has been tested as a substitute for cisplatin in combination chemotherapy with similar results ^13, 15^. The use of oxaliplatin can be recommended for patients suffering from renal insufficiency or patients with severe adverse events after cisplatin treatment ^8^. Irinotecan or Docetaxel combinations have not been established for the perioperative treatment of gastric cancer in 2004. Both drugs were used in combinations for palliative therapy only ^16, 17^.

At the ASCO meeting in 2011 the FLOT3 trial ^15^ has been presented:

Patients with untreated operable gastric adenocarcinoma received four preoperative cycles of FLOT (5-FU, leucovorin, oxaliplatin, docetaxel) and underwent surgical resection (D2 resection) followed by four postoperative cycles. Patients with limited metastatic disease (distant intra-abdominal lymph nodes and/or a maximum of one organ involved in metastatic disease, ECOG-PS < 1) received eight cycles and underwent surgical resection when a complete macroscopic resection seemed possible. Surgical treatment was conducted in 95.7% and 42.64% of the patients, respectively. For operable patients the median overall survival was not reached, for patients with limited disease the median overall survival was 18.6 months; there were also significant differences in progression-free survival (p < 0.001). Grade 3–4 toxicities were similar among the groups and occurred in 70.6% *vs.* 72.1% of the patients, respectively ^15^.

In gastric adenocarcinoma or adenocarcinoma of the gastrooesophageal junction stage uT2 a perioperative chemotherapy can be considered (Level of evidence 1b), in stage uT3 and in resectable uT4a adenocarcinomas patients should receive a pre- and postoperative polychemotherapy (Level of evidence 1b) ^8^.

Our patient may be one of the rare cases with advanced gastric cancer cured by perioperative third line chemotherapy and surgery.

## Consent

Written informed consent for publication of clinical details and clinical images was obtained from the patient.
