# Supplementary material for: Curative treatment in a patient with gastric cancer stage IV: a case report
Source: F1000Res. 2012 Oct 19;1:34. [Version 1] doi: 10.12688/f1000research.1-34.v1 (PMC3782351; doi:10.12688/f1000research.1-34.v1)
Supplement: Peer review history

# Peer review: Referee response for version 1

**Published:** 2012-11-19

**Authors:** Peter M Schlag

**Affiliations:** 1 Charité Comprehensive Cancer Center, Charité Campus, Berlin, Germany

This is a single case report without any new information and is without any scientific background.

I have read this submission. I believe that I have an appropriate level of expertise to state that I do not consider it to be of an acceptable scientific standard, for reasons outlined above.

# Peer review: Referee response for version 1

**Published:** 2012-11-14

**Authors:** S Yousuf Zafar

**Affiliations:** 1 Department of Medicine, Duke University Medical Center, Durham, NC, USA

I have read this submission. I believe that I have an appropriate level of expertise to confirm that it is of an acceptable scientific standard.

# Peer review: Referee response for version 1

**Published:** 2012-11-09

**Authors:** Ian Beales

**Affiliations:** 1 School of Health Policy and Practice, University of East Anglia, Norwich, Norfolk, UK

The authors do discuss the FLOT3 trial at disproportionate detail compared to the rest of the discussion and probably a bit too excessively in response to the case reported. Whilst these data are interesting, it would be helpful to place these in a bit more context in relation to the case reported.

There are some typographical and grammatical errors in the Discussion section (pre-operative and insufficient data in the first paragraph).

The discussion about irinotecan and docetaxel combinations in the Discussion section: the authors could clarify whether they are primarily discussing the situation of current knowledge or as it stood in 2004. It would be most appropriate to discuss the current state of this combination in the article.

I have read this submission. I believe that I have an appropriate level of expertise to confirm that it is of an acceptable scientific standard.

# Peer review: Referee response for version 1

**Published:** 2012-11-08

**Authors:** Maria Alsina Maqueda

**Affiliations:** 1 Department of Medical Oncology, Medical Oncology, University Hospital Vall d'Hebron, Universitat Autònoma de Barcelona, Barcelona, Spain

Overall, this case report is interesting and worthy of publication. There are however a few revisions that should be made:

1. In the Introduction section (2nd paragraph, 1st sentence), the chemotherapy scheme used in the patient is not the same as the one used by the study referenced; the scheme should be justified.

2. In the Introduction section (3rd paragraph, last sentence) there are more recent references to justify the classification statement ( Thuss-Patiente *Eur J* Cancer 2011, Kang *Clin Oncol* 2012 and Van Cutsem ASCO GI 2012).

3. In the Discussion section (4th & 5th paragraphs) the authors refer to a study published in ASCO/JCO 2008, not 2011 which they have stated.

4.In the Discussion section (5th paragraph) the authors describe a study extensively with compared with the rest of the article, maybe authors should justify why (the use of docetaxel?).

5. The authors should discuss more extensively the use of the three lines with different efficacies in the neoadjuvant setting, and they should also address the issue of long gastric patient survivors.

6. In the Reference section, references 9 and 10 references refer to the same study; it is the newest one should appear, and the older one can be removed.

I have read this submission. I believe that I have an appropriate level of expertise to confirm that it is of an acceptable scientific standard.
